# Influence of primary payer status on the management and outcomes of ST-segment elevation myocardial infarction in the United States

**DOI:** 10.1371/journal.pone.0243810

**Published:** 2020-12-18

**Authors:** Saraschandra Vallabhajosyula, Vinayak Kumar, Pranathi R. Sundaragiri, Wisit Cheungpasitporn, Malcolm R. Bell, Mandeep Singh, Allan S. Jaffe, Gregory W. Barsness

**Affiliations:** 1 Department of Cardiovascular Medicine, Mayo Clinic, Rochester, Minnesota, United States of America; 2 Division of Pulmonary and Critical Care Medicine, Department of Medicine, Mayo Clinic, Rochester, Minnesota, United States of America; 3 Center for Clinical and Translational Science, Mayo Clinic Graduate School of Biomedical Sciences, Rochester, Minnesota, United States of America; 4 Section of Interventional Cardiology, Division of Cardiovascular Medicine, Department of Medicine, Emory University School of Medicine, Atlanta, Georgia, United States of America; 5 Department of Medicine, Mayo Clinic, Rochester, Minnesota, United States of America; 6 Division of Hospital Internal Medicine, Department of Medicine, Mayo Clinic, Rochester, Minnesota, United States of America; 7 Division of Nephrology, Department of Medicine, University of Mississippi School of Medicine, Jackson, Mississippi, United States of America; IRCCS Policlinico S.Donato, ITALY

## Abstract

**Background:**

There are limited contemporary data on the influence of primary payer status on the management and outcomes of ST-segment elevation myocardial infarction (STEMI).

**Objective:**

To assess the influence of insurance status on STEMI outcomes.

**Methods:**

Adult (>18 years) STEMI admissions were identified using the National Inpatient Sample database (2000–2017). Expected primary payer was classified into Medicare, Medicaid, private, uninsured and others. Outcomes of interest included in-hospital mortality, use of coronary angiography and percutaneous coronary intervention (PCI), hospitalization costs, hospital length of stay and discharge disposition.

**Results:**

Of the 4,310,703 STEMI admissions, Medicare, Medicaid, private, uninsured and other insurances were noted in 49.0%, 6.3%, 34.4%, 7.2% and 3.1%, respectively. Compared to the others, the Medicare cohort was older (75 vs. 53–57 years), more often female (46% vs. 20–36%), of white race, and with higher comorbidity (all *p*<0.001). The Medicare and Medicaid population had higher rates of cardiogenic shock and cardiac arrest. The Medicare cohort had higher in-hospital mortality (14.2%) compared to the other groups (4.1–6.7%), p<0.001. In a multivariable analysis (Medicare referent), in-hospital mortality was higher in uninsured (adjusted odds ratio (aOR) 1.14 [95% confidence interval {CI} 1.11–1.16]), and lower in Medicaid (aOR 0.96 [95% CI 0.94–0.99]; *p* = 0.002), privately insured (aOR 0.73 [95% CI 0.72–0.75]) and other insurance (aOR 0.91 [95% CI 0.88–0.94]); all *p*<0.001. Coronary angiography (60% vs. 77–82%) and PCI (45% vs. 63–70%) were used less frequently in the Medicare population compared to others. The Medicare and Medicaid populations had longer lengths of hospital stay, and the Medicare population had the lowest hospitalization costs and fewer discharges to home.

**Conclusions:**

Compared to other types of primary payers, STEMI admissions with Medicare insurance had lower use of coronary angiography and PCI, and higher in-hospital mortality.

## Introduction

Ischemic heart disease and specifically acute myocardial infarction continue to be leading cause of cardiovascular admissions in the United States [1–4]. ST-segment elevation myocardial infarction (STEMI) constitutes about 30% of all acute myocardial infarction admissions in the United Sates. Despite the improvements in early coronary angiography and prompt percutaneous coronary intervention (PCI), STEMI continues to be associated with considerable mortality and morbidity. In addition, STEMI is associated with high resource utilization with estimated costs of hospitalization of United States Dollars 21,000–26,000 [5].

In the United States, prior studies in cardiac and non-cardiac patient populations have demonstrated that insurance status may have an association with clinical outcomes [6–9]. In patients with STEMI, large retrospective studies have previously demonstrated the lack of health insurance and Medicaid status to be associated with worse mortality and higher readmission rates compared to patients with private insurance [7, 10, 11]. While this may be partially be due to socioeconomic factors, other contributing factors include lifestyle issues, a lack of access to care in uninsured populations, and a higher burden of comorbidities and issues with medication adherence in Medicaid populations that preclude PCI [7, 10]. As of 2018, approximately 8.5% of people in the US are uninsured, and of those who have insurance, approximately 34% have a public plan such as Medicare and Medicaid [12]. As the population in the United States grows older and has higher comorbidity, cardiovascular emergencies continue to be a leading health care issue [13].

In the current era, it is unclear if insurance status continues to play a role in the management and outcomes of STEMI [10]. Using an 18-year national database, we sought to assess the management and outcomes of STEMI by primary payer status. In addition, we evaluated the temporal trends in admissions, use of cardiac procedures, and clinical outcomes of these populations.

## Material and methods

### Study population, variables and outcomes

The National (Nationwide) Inpatient Sample (NIS) is the largest all-payer database of hospital inpatient stays in the United States. NIS contains discharge data from a 20% stratified sample of community hospitals and is a part of the Healthcare Quality and Utilization Project (HCUP), sponsored by the Agency for Healthcare Research and Quality [14]. Information regarding each discharge includes patient demographics, primary payer, hospital characteristics, principal diagnosis, up to 24 secondary diagnoses, and procedural diagnoses. The HCUP-NIS does not capture individual patients but captures all information for a given admission. Institutional Review Board approval was not sought due to the publicly available nature of this de-identified database. These data are available to other authors via the HCUP-NIS database with the Agency for Healthcare Research and Quality [14].

Using the HCUP-NIS data from 2000–2017, a retrospective cohort study of adult admissions (≥18 years of age) with STEMI in the primary diagnosis field (International Classification of Diseases 9.0 Clinical Modification [ICD-9CM] 410.1x-410.6x, 410.8x, 410.9x and ICD-10CM I21.x-22.x except I21.4, I 32.Ax, I22.2, and I21.9) was identified [4]. The HCUP-NIS contains data on the expected primary payers as Medicare (includes fee-for-service and Medicare Advantage), Medicaid (includes fee-for-service and managed care), private insurance (Blue Cross, commercial carriers, private health maintenance organizations and preferred provider organizations), uninsured (self-pay or no charge) and others (includes Worker’s Compensation, CHAMPUS, CHAMPVA, Title V and other government programs) [7, 10]. Medicare primary serves adults over 65 years age and younger disabled and dialysis patients. Medicaid is a primary assistance program for low-income people of all ages and patients typically pay no part of the costs. Admissions with missing primary payer category and in-hospital mortality were excluded. The Deyo’s modification of the Charlson Comorbidity Index was used to identify the burden of co-morbid diseases (**S1 Table**) [15]. Demographic characteristics, hospital characteristics, complications, acute organ failure, cardiac procedures, and non-cardiac procedures were identified for all admissions using previously used methodologies from our group [1–4, 13, 16–39].

The primary outcome was the in-hospital mortality in STEMI admissions stratified by insurance status. Secondary outcomes included temporal trends in admissions, use of coronary angiography, early coronary angiography, percutaneous coronary intervention (PCI), coronary artery bypass grafting (CABG), mechanical circulatory support, hospitalization costs, length of hospital stay and discharge disposition.

### Statistical analysis

As recommended by HCUP-NIS, survey procedures using discharge weights provided with HCUP-NIS database were used to generate national estimates [40]. Using the trend weights provided by the HCUP-NIS, samples from 2000–2011 were re-weighted to adjust for the 2012 HCUP-NIS re-design [40]. One-way analysis of variance and t-tests were used to compare categorical and continuous variables, respectively. Multivariable logistic regression was used to analyze trends over time (referent year 2000). Univariable analysis for trends and outcomes was performed and was represented as odds ratio (OR) with 95% confidence interval (CI). Multivariable logistic regression analysis incorporating age, sex, race, socio-economic stratum, hospital characteristics, comorbidities, year of admission, STEMI location, cardiogenic shock, cardiac arrest, do-not-resuscitate (DNR) status and palliative care referral was performed for assessing coronary angiography and temporal trends of coronary angiography. Multivariable logistic regression analysis incorporating age, sex, race, socio-economic stratum, hospital characteristics, comorbidities, year of admission, STEMI location, cardiogenic shock, cardiac arrest, acute respiratory failure, acute kidney injury, systolic heart failure, prior CABG, complications, cardiac procedures, non-cardiac procedures, DNR status and palliative care referral was performed for assessing in-hospital mortality and temporal trends of in-hospital mortality. For the multivariable modeling, regression analysis with purposeful selection of statistically (liberal threshold of *p*<0.20 in univariate analysis) and clinically relevant variables was conducted. To confirm the results of the primary analysis, sensitivity analyses were performed stratifying the population by age (≤/> 75 years), sex, race (white/non-white), tertiles of study period, use of PCI and use of DNR status/palliative care referral.

The inherent restrictions of the HCUP-NIS database related to research design, data interpretation, and data analysis were reviewed and addressed [40, 41]. Pertinent considerations include not assessing individual hospital-level volumes (due to changes to sampling design detailed above), treating each entry as an ‘admission’ as opposed to individual patients, restricting the study details to inpatient factors since the HCUP-NIS does not include outpatient data, and limiting administrative codes to those previously validated and used for similar studies. Two-tailed *p*<0.05 was considered statistically significant. All statistical analyses were performed using SPSS v25.0 (IBM Corp, Armonk NY).

## Results

In the period from January 1, 2000 to December 31, 2017, there were 4,320,097 STEMI admissions, of which primary payer status could not be ascertained in 9,394 (0.2%). In the final cohort of 4,310,703 STEMI admissions, Medicare, Medicaid, Private, Uninsured and Others constituted 2,113,356 (49.0%), 269,507 (6.3%), 1,484,385 (34.4%), 311,773 (7.2%) and 131,682 (3.1%), respectively. The Medicare cohort was significantly older than other groups, with higher prevalence of females, white race, and higher comorbidity (all *p*<0.001) (**Table 1**). The Medicare cohort had lower rates of inferior STEMI (39.2%) as compared to the rest (43.5–49.5%) (*p*<0.001). The Medicare and Medicaid population had higher rates of cardiogenic shock, cardiac arrest, respiratory failure, acute kidney injury and complication rates compared to the other groups (**Table 1**).

**Table 1:** Characteristics of STEMI admissions stratified by primary payer status.

Characteristics		Medicare	Medicaid	Private	Uninsured	Others	*P*
		(N = 2,113,356)	(N = 269,507)	(N = 1,484,385)	(N = 311,773)	(N = 131,682)	
**Age (years)**		75.2 ± 10.4	54.0 ± 11.1	56.0 ± 10.2	52.5 ± 9.7	57.0 ± 11.5	<0.001
**Female sex**		46.2	36.1	23.4	23.9	20.4	<0.001
**Weekend admission**		25.8	27.2	27.6	27.4	26.6	<0.001
**Race**	**White**	66.0	47.9	63.0	55.0	55.9	<0.001
	**Black**	5.4	12.4	5.1	9.4	7.7	
	**Others**^**a**^	28.6	39.7	31.9	35.7	36.4	
**Quartile of median household income**	**0-25**^**th**^ **percentile**	22.9	34.9	16.5	31.1	26.4	<0.001
	**26**^**th**^**-50**^**th**^ **percentile**	28.6	28.9	24.8	29.2	28.8	
	**51**^**st**^**-75**^**th**^ **percentile**	24.7	21.2	26.8	23.3	24.8	
	**75**^**th**^**-100**^**th**^ **percentile**	23.8	14.9	31.9	16.4	20.0	
**Charlson Comorbidity Index**	**0–3**	23.5	75.4	79.6	85.6	75.0	<0.001
	**4–6**	58.4	21.1	17.9	13.1	21.3	
	**≥ 7**	18.1	3.6	2.5	1.3	3.7	
**Hospital teaching status and location**	**Rural**	16.0	9.6	7.8	10.1	12.2	<0.001
	**Urban non-teaching**	40.5	34.6	40.7	40.3	39.4	
	**Urban teaching**	43.5	55.8	51.5	49.6	48.3	
**Hospital bed-size**	**Small**	12.4	9.3	9.4	9.1	8.1	<0.001
	**Medium**	25.1	24.5	24.6	24.0	24.0	
	**Large**	62.5	66.2	66.1	66.8	67.9	
**Hospital region**	**Northeast**	18.6	21.4	18.6	11.6	9.0	<0.001
	**Midwest**	24.5	20.7	24.4	19.1	18.1	
	**South**	39.2	33.4	37.7	55.2	46.9	
	**West**	17.7	24.5	19.4	14.1	25.9	
**STEMI location**	**Anterior**	31.1	34.6	33.3	34.5	33.2	<0.001
	**Inferior**	39.2	43.5	49.5	48.0	46.9	<0.001
	**Other**	29.3	21.4	17.1	17.4	19.1	<0.001
**Cardiogenic shock**		9.9	9.8	6.7	7.4	7.3	<0.001
**Cardiac arrest**		9.2	10.0	8.4	9.2	8.6	<0.001
**Acute respiratory failure**		11.2	11.0	6.5	7.6	7.7	<0.001
**Acute kidney injury**		11.9	9.2	5.1	5.6	6.6	<0.001
**Systolic heart failure**		5.4	6.2	3.1	3.9	4.0	<0.001
**Vascular complications**		1.0	0.9	0.9	0.7	0.9	<0.001
**Hemorrhage**		1.9	1.7	1.2	1.1	1.1	<0.001
**Ischemic stroke**		2.0	1.4	0.8	0.8	0.9	<0.001
**Intracranial hemorrhage**		0.4	0.3	0.2	0.2	0.2	<0.001
**Intravascular ultrasound**		1.2	2.3	2.0	2.0	1.7	<0.001
**Coronary thrombectomy**		0.9	2.0	1.3	1.3	1.4	<0.001
**Pulmonary artery catheterization**		1.5	1.5	1.2	1.1	1.2	<0.001
**Invasive mechanical ventilation**		10.1	10.6	6.2	7.5	7.2	<0.001
**Hemodialysis**		0.6	0.6	0.3	0.3	0.3	<0.001

Represented as percentage or mean ± standard deviation; ^a^Hispanic, Asian, Native American, Others

**Abbreviations:** STEMI: ST-segment-elevation myocardial infarction

**Fig 1:**
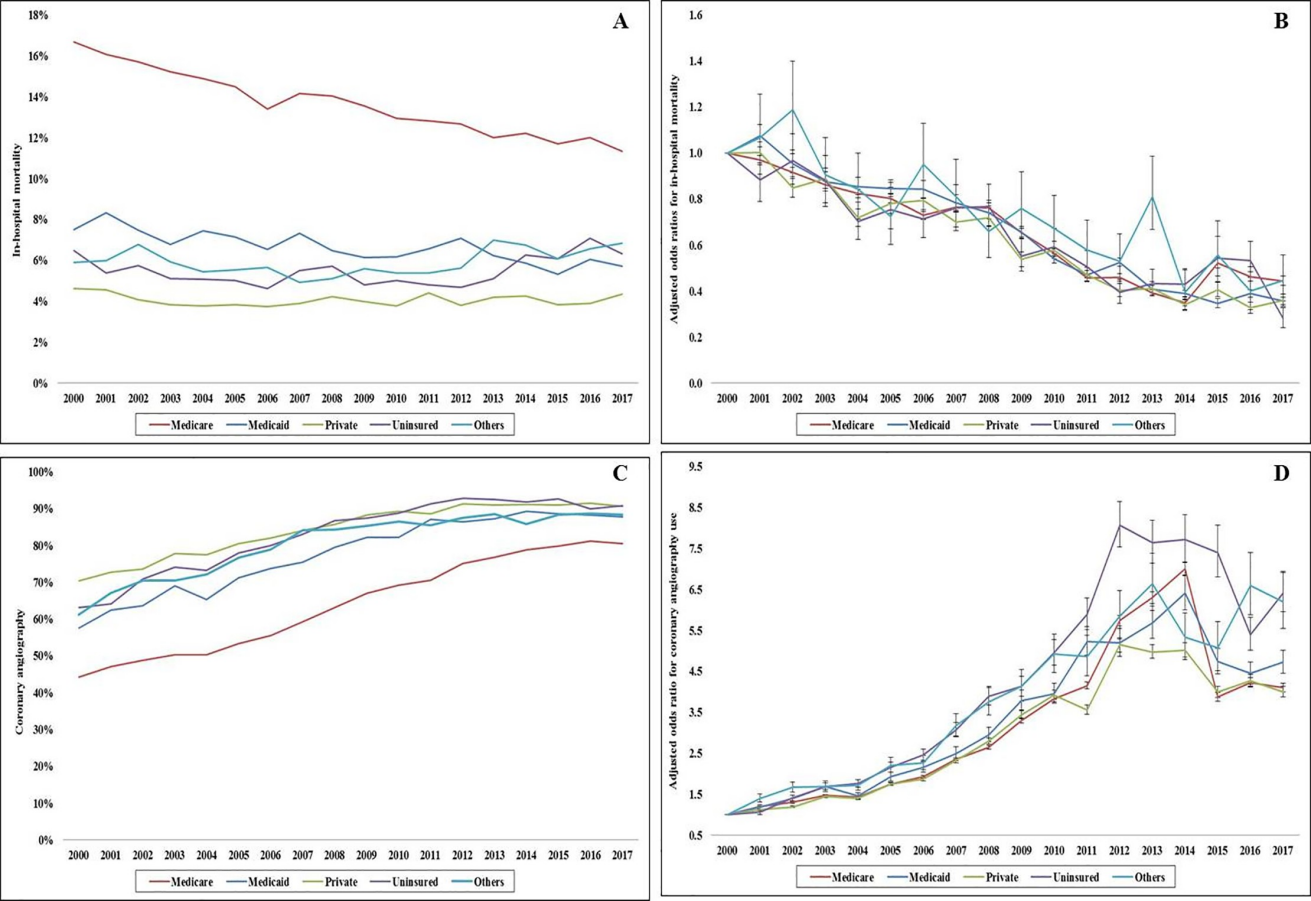
Temporal trends of coronary angiography use and in-hospital mortality in STEMI admissions stratified by insurance status. **A**: 18-year unadjusted trends of in-hospital mortality in STEMI admissions; *p*<0.001 for trend over time; **B**: Adjusted multivariate logistic regression for temporal trends of in-hospital mortality in STEMI admissions (2000 as referent year)*; *p*<0.001 for trend over time; **C:** 18-year unadjusted trends of coronary angiography use in STEMI; all *p*<0.001 for trend over time; **D**: Adjusted multivariate logistic regression for temporal trends of coronary angiography use in STEMI admissions (2000 as referent year)**; *p*<0.001 for trend over time; *Adjusted for age, sex, race, socio-economic stratum, hospital characteristics, comorbidities, year of admission, STEMI location, cardiogenic shock, cardiac arrest, acute respiratory failure, acute kidney injury, systolic heart failure, prior CABG, complications, cardiac procedures, non-cardiac procedures, DNR status and palliative care referral; **Adjusted for age, sex, race, socio-economic stratum, hospital characteristics, comorbidities, year of admission, STEMI location, cardiogenic shock, cardiac arrest, do-not-resuscitate (DNR) status and palliative care referral; **Abbreviations:** STEMI: ST-segment elevation myocardial infarction.

**Fig 2:**
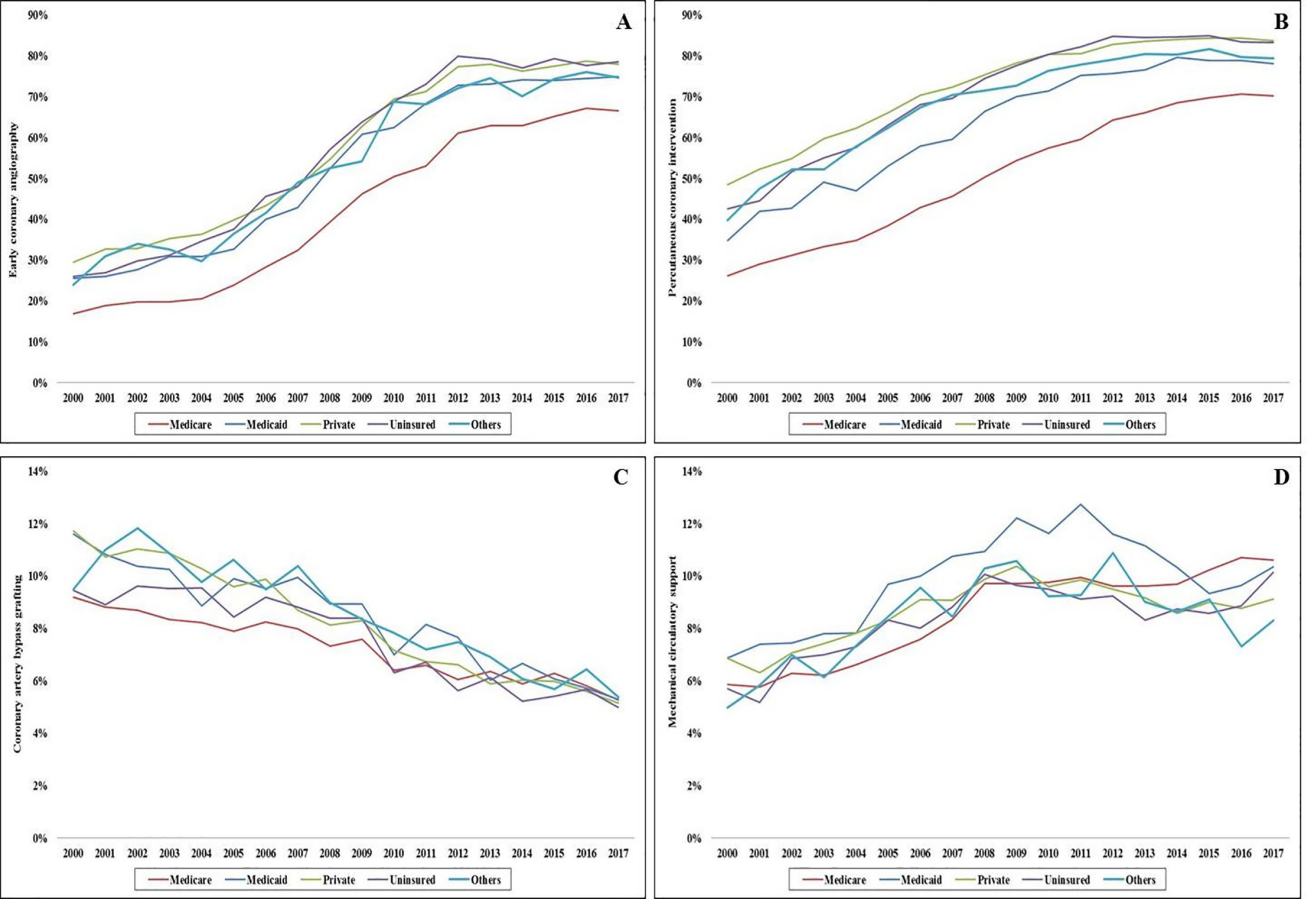
Temporal trends in cardiac procedures use in STEMI admissions stratified by insurance status. Eighteen-year trends of early coronary angiography (**A**), percutaneous coronary intervention (**B**), coronary artery bypass grafting (**C**), and mechanical circulatory support (**D**) in STEMI admissions stratified by insurance status; all *p*<0.001 for trend.

Coronary angiography was used less frequently in the Medicare population (59.6%) compared to the other cohorts (77.4–82.2%) during this 18-year period. There was a steady increase in the use of coronary angiography across all insurance sub-groups; however the Medicare cohort had consistently lower use (**Fig 1A**). In adjusted analyses, compared to 2000, all primary payer cohorts showed a 5–6 fold increase in the use of coronary angiography in 2017 (**Fig 1B**). In a multivariable logistic regression analysis with Medicare population as referent category, coronary angiography was used less often in the Medicaid population (OR 0.93 [0.91–0.94]), and more often in all other populations–private insurance (OR 1.25 [95% CI 1.24–1.26]), uninsured (OR 1.09 [95% CI 1.07–1.10]) and other insurance (OR 1.14 [95% CI 1.12–1.16]); all *p*<0.001 (**S2 Table**). Early coronary angiography and PCI were performed less frequently in the Medicare population, and CABG was performed less frequently in the Medicare and uninsured admissions (**Table 2**). During this 18-year period, there was a steady increase in early coronary angiography and PCI use across all 5 cohorts; however the Medicare group had consistently lower utilization (**Fig 2A and 2B**). The Medicaid cohort had higher rates of mechanical circulatory support use compared to the other groups (**Table 2**). There was a steady decline in CABG use across all sub-groups and an initial peak till 2009 followed by a decline in mechanical circulatory support use across all cohorts (**Fig 2C and 2D**).

**Table 2:** Clinical outcomes of STEMI admissions stratified by primary payer status.

Outcomes		Medicare	Medicaid	Private	Uninsured	Others	*P*
		(N = 2,113,356)	(N = 269,507)	(N = 1,484,385)	(N = 311,773)	(N = 131,682)	
**In-hospital mortality**		14.2	6.7	4.1	5.4	5.9	<0.001
**Coronary angiography**		59.6	77.4	82.2	82.2	79.3	<0.001
**Early coronary angiography (day 0)**		35.6	52.4	51.4	54.7	50.4	<0.001
**Percutaneous coronary intervention**		45.3	62.9	68.9	69.7	66.2	<0.001
**Coronary artery bypass grafting**		7.7	8.4	8.8	7.7	8.9	<0.001
**Mechanical circulatory support**	**Total**	7.9	9.7	8.4	8.2	8.2	<0.001
	**IABP**	7.6	9.2	8.1	7.9	7.9	<0.001
	**pLVAD**	0.3	0.5	0.3	0.3	0.3	<0.001
	**ECMO**	0.1	0.2	0.1	0.1	0.1	<0.001
**Palliative care referral**		1.9	1.0	0.6	0.6	1.7	<0.001
**Do-not-resuscitate status**		2.7	1.1	0.5	0.7	1.1	<0.001
**Length of stay (days)**		5.5 ± 6.3	5.5 ± 9.7	4.2 ± 5.0	4.2 ± 5.2	4.4 ± 5.3	<0.001
**Hospitalization costs (x1000 USD)**		59 ± 79	79 ± 111	63 ± 77	65 ± 69	66 ± 83	<0.001
**Disposition**	**Home**	55.6	74.7	79.5	83.2	78.8	<0.001
	**Transfer**	13.5	10.4	12.0	10.0	11.7	
	**Skilled nursing facility**	18.5	5.6	3.3	1.8	4.4	
	**Home with HHC**	11.8	6.8	4.7	2.8	3.9	
	**Against medical advice**	0.6	2.5	0.4	2.3	1.2	

Represented as percentage or mean ± standard deviation

**Abbreviations:** ECMO: extracorporeal membrane oxygenation; HHC: home health care; IABP: intra-aortic balloon pump; pLVAD: percutaneous left ventricular assist device; STEMI: ST-segment-elevation myocardial infarction; USD: United States Dollars

The unadjusted all-cause in-hospital mortality was the highest in the Medicare cohort (14.2%) compared to the other groups (4.1–6.7%) (**Table 2**). The Medicare population had consistently higher in-hospital mortality during the 18-year study period, despite a comparable decline in 2017 compared to 2000 across all cohorts (**Fig 1C and 1D**). In a multivariable logistic regression analysis with Medicare as the referent population, in-hospital mortality was higher in the uninsured (OR 1.14 [95% CI 1.11–1.16]), and lower in all other populations–Medicaid (OR 0.96 [95% CI 0.94–0.99]; *p* = 0.002), privately insured (OR 0.73 [95% CI 0.72–0.75]) and other insurance (OR 0.91 [95% CI 0.88–0.94]); all *p*<0.001 (**S3 Table**).

Multiple sensitivity analyses were performed to confirm the results of the primary findings. The primary results were consistent in all sub-groups except those aged >75 years and with use of palliative care referral and DNR status use (**Table 3**). The Medicare and Medicaid populations had longer lengths of hospital stay, and the Medicare population had the least hospitalization costs and fewer discharges to home (**Table 2**). Palliative care and DNR status use were low (<3%) with the highest rates in the Medicare population (**Table 2**).

**Table 3:** In-hospital mortality in STEMI admissions stratified by patient characteristics.

Patient characteristics^a^			Odds ratio	95% confidence interval		*P*
				Lower Limit	Upper Limit	
**Age group**	**≤75 years**	**Medicare**	Reference category			
		**Medicaid**	0.69	0.67	0.71	<0.001
		**Private**	0.54	0.54	0.55	<0.001
		**Uninsured**	0.84	0.82	0.86	<0.001
		**Others**	0.64	0.62	0.67	<0.001
	**>75 years**	**Medicare**	Reference category			
		**Medicaid**	0.91	0.86	0.97	0.002
		**Private**	1.03	1.00	1.05	0.048
		**Uninsured**	1.14	1.05	1.23	0.001
		**Others**	1.26	1.19	1.34	0.000
**Sex**	**Male**	**Medicare**	Reference category			
		**Medicaid**	0.75	0.72	0.77	<0.001
		**Private**	0.60	0.59	0.61	<0.001
		**Uninsured**	0.91	0.89	0.94	<0.001
		**Others**	0.75	0.72	0.78	<0.001
	**Female**	**Medicare**	Reference category			
		**Medicaid**	0.77	0.75	0.80	<0.001
		**Private**	0.67	0.66	0.69	<0.001
		**Uninsured**	0.87	0.84	0.91	<0.001
		**Others**	0.88	0.84	0.94	<0.001
**Race**	**White**	**Medicare**	Reference category			
		**Medicaid**	0.75	0.72	0.77	<0.001
		**Private**	0.61	0.60	0.62	<0.001
		**Uninsured**	0.86	0.83	0.89	<0.001
		**Others**	0.77	0.74	0.80	<0.001
	**Non-white**^**b**^	**Medicare**	Reference category			
		**Medicaid**	0.77	0.74	0.79	<0.001
		**Private**	0.64	0.63	0.66	<0.001
		**Uninsured**	0.93	0.90	0.96	<0.001
		**Others**	0.81	0.77	0.85	<0.001
**Tertiles of study period**	**2000–2005**	**Medicare**	Reference category			
		**Medicaid**	0.84	0.81	0.86	<0.001
		**Private**	0.65	0.64	0.67	<0.001
		**Uninsured**	0.90	0.87	0.94	<0.001
		**Others**	0.78	0.74	0.81	<0.001
	**2006–2011**	**Medicare**	Reference category			
		**Medicaid**	0.80	0.77	0.83	<0.001
		**Private**	0.67	0.66	0.69	<0.001
		**Uninsured**	0.96	0.92	1.00	0.06
		**Others**	0.81	0.77	0.86	<0.001
	**2012–2017**	**Medicare**	Reference category			
		**Medicaid**	0.67	0.64	0.69	<0.001
		**Private**	0.57	0.56	0.59	<0.001
		**Uninsured**	0.89	0.85	0.93	<0.001
		**Others**	0.80	0.75	0.85	<0.001
**Percutaneous coronary intervention**	**Yes**	**Medicare**	Reference category			
		**Medicaid**	0.73	0.71	0.76	<0.001
		**Private**	0.54	0.53	0.55	<0.001
		**Uninsured**	0.87	0.84	0.90	<0.001
		**Others**	0.65	0.62	0.69	<0.001
	**No**	**Medicare**	Reference category			
		**Medicaid**	0.77	0.75	0.79	<0.001
		**Private**	0.70	0.68	0.71	<0.001
		**Uninsured**	0.90	0.88	0.93	<0.001
		**Others**	0.89	0.85	0.92	<0.001
**Palliative care or do-not-resuscitate status**	**Yes**	**Medicare**	Reference category			
		**Medicaid**	0.94	0.86	1.02	0.11
		**Private**	1.38	1.31	1.45	<0.001
		**Uninsured**	1.47	1.32	1.63	<0.001
		**Others**	1.59	1.45	1.74	<0.001
	**No**	**Medicare**	Reference category			
		**Medicaid**	0.75	0.74	0.77	<0.001
		**Private**	0.60	0.59	0.61	<0.001
		**Uninsured**	0.88	0.86	0.90	<0.001
		**Others**	0.74	0.72	0.77	<0.001

^**a**^Each sub-group was adjusted for age, sex, race, socio-economic stratum, hospital characteristics, comorbidities, year of admission, STEMI location, cardiogenic shock, cardiac arrest, acute respiratory failure, acute kidney injury, systolic heart failure, prior CABG, complications, cardiac procedures, non-cardiac procedures, DNR status and palliative care referral

^b^Black, Hispanic, Asian, Native American, Others

## Discussion

In the largest study looking at the influence of primary payer status on the outcomes of STEMI, we noted that the Medicare beneficiaries differed significantly in age, socio-demographic characteristics, and their clinical course. The Medicare beneficiaries consistently received less frequent guideline-directed procedures, had higher rates of in-hospital complications and worse in-hospital outcomes. Though age may partly explain these differences, there remain significant differences between the various insurance categories.

The data from this study are consistent with prior work from the HCUP-NIS database as well as other databases that have evaluated insurance-based disparities in STEMI care. A 2019 study by Patel *et al* used the HCUP-NIS database from 2012–2015 to compare STEMI outcomes in Medicaid and private insurance patients in a propensity-matched analysis, finding that there were higher rates of revascularization, lower in-hospital mortality, and a lower cost of hospitalization in patients with private insurance [7]. The authors provide several potential explanations for these findings including residual confounders despite propensity matching and provider reluctance to initiate dual antiplatelet therapy due to concerns for medication adherence. A 2018 study by Niedzwiecki *et al* that used AMI data from the California Office of State Health Planning and Development from 2001–2014, found that Medicaid populations, which had a disproportionately higher mix of black and Hispanic populations and higher prevalence of peripheral vascular disease, pulmonary disease, diabetes mellitus, and renal failure, had lower rates of PCI than privately insured and uninsured populations, with a significantly higher mortality at 30 days, 90 days, and 1 year [6]. In addition, these insurance categories further reinforce racial disparities in health care access and delivery. Notably, these outcomes held true even when the study was limited to STEMI populations, despite patients having a high likelihood of being admitted to PCI-capable hospitals. The authors hypothesized that financial factors may play a role as Medicaid reimbursement may be lower than that from uninsured population, as Medicaid rates in California are among the lowest in the United States. Similarly, a 2017 study by Pancholy *et al*, the authors use HCUP-NIS 2003–2014 database to study the effect of insurance status on the adult STEMI population [10]. In that study, the Medicare population had lower rates of coronary intervention than the other insurance categories, with higher rates of numerous in-hospital outcomes such as cardiogenic shock, pneumonia, gastrointestinal hemorrhage, acute stroke, and increased length of stay. Furthermore, vulnerable populations, such as older adults and racial minorities have poor awareness of symptoms and experience delays in seeking health care which may contribute to poorer outcomes in these populations [42, 43]. The lack of insurance was an independent risk factor for higher in-hospital mortality despite lower incidence of comorbidities, for which the authors suggest that the uninsured group likely has unrecognized disease associated with poor lifestyle choices and lack of preventative medicine [42, 43].

As noted in this study, the Medicare population appears to have consistently higher rates of in-hospital mortality than the other insurance groups, though this difference is declining over time. A large part of this is due to the intrinsic differences between the populations bearing Medicare versus other insurance types–the Medicare population is older, with higher comorbidity, and greater severity of illness [10]. However, importantly, the Medicare population consistently received lower rates of coronary angiography and PCI despite robust clinical guidelines [44]. The insurance-based disparities were prominent in those aged ≤75 years but not in older adults. Despite prior data showing the value of PCI in older adults, this study continues to note a lower use of PCI in this population [13, 45]. Despite being adjusted for comorbidities and acuity, the Medicare population continued to have worse in-hospital outcomes. This study that shows that Medicare and Medicaid populations had higher rates of invasive mechanical ventilation and hemodialysis compared to the other insurance populations, suggesting that the choice to reduce PCI usage in Medicare populations may have been a risk-avoidance strategy or the refusal of advanced care in older patients. While Medicaid populations seem to have relatively similar rates of these complications, the overall mortality is much lower than in Medicare populations, which is likely a function of being younger with lower comorbidity. This is consistent with the literature, as cardiovascular, pulmonary, and renal comorbidities have been associated with worse outcomes after STEMI [22, 23, 25, 26, 30]. Notably, in the current study, the Medicare population has lower hospitalization costs, possibly related to the lower coronary angiography rates, more frequent use of palliative care referral, higher rates of do-not-resuscitate status, and higher in-hospital mortality compared to patients with private insurance. It has been previously shown that older patients were more likely to have elected for DNR status, even when controlling for severity of illness, compared to younger populations [46]. Moreover, prior studies have demonstrated that DNR status was associated with higher mortality rates in patients with AMI, which is consistent with the results seen in this analysis [47]. Therefore, in this study and others, it appears that insurance status is closely related to social, economic, and environmental factors that need to be considered in future qualitative studies.

### Limitations

This study has several limitations, some of which are inherent to the analysis of a large administrative database. The HCUP-NIS attempts to mitigate potential errors by using internal and external quality control measures. Insurance status is frequently associated with other socio-economic categories, which cannot be fully evaluated using an administrative database. The lack of angiographic data, such PCI location, lesion classification, presence of multi-vessel disease, and revascularization failure, that may significantly influence outcomes, were not available in this database. There are limited data on patient and family specific limitations to therapeutic options which may influence the clinical outcomes in this population. This study does not study post-hospital long-term complications after PCI and STEMI, which may result in additional health care utilization that might be challenging for vulnerable populations. Additionally, this study does not study an exhaustive list of comorbidities that may contribute to outcomes, such as frailty, which may confound the results seen here [48]. Despite these limitations, this study addresses a significant knowledge gap highlighting the clinical outcomes of STEMI when evaluated using an insurance perspective.

## Conclusions

Compared to other types of primary payers, STEMI admissions with Medicare coverage have lower use of guideline-directed procedures, have higher rates of complications, and worse in-hospital outcomes. Further data are needed to understand the complex socio-demographic underpinnings associated with insurance coverage which may determine quality of care and outcomes in this acutely ill population.

## Supporting information

S1 Table Administrative codes. (DOCX)

S2 Table Predictors of coronary angiography use in STEMI. (DOCX)

S3 Table Predictors of in-hospital mortality in STEMI. (DOCX)

## Abbreviations

CABG: coronary artery bypass grafting
CI: confidence interval
DNR: do-not-resuscitate
HCUP: Healthcare Cost and Utilization Project
ICD-9CM: International Classification of Diseases-9 Clinical Modification
ICD-10CM: International Classification of Diseases-10 Clinical Modification
NIS: National/Nationwide Inpatient Sample
OR: odds ratio
PCI: percutaneous coronary intervention
STEMI: ST-segment elevation myocardial infarction
