# Supplementary material for: Influence of primary payer status on the management and outcomes of ST-segment elevation myocardial infarction in the United States
Source: PLoS One. 2020 Dec 18;15(12):e0243810. doi: 10.1371/journal.pone.0243810 (PMC7748387; doi:10.1371/journal.pone.0243810)
Supplement: S1 Table — (DOCX) [file pone.0243810.s001.docx]

**S1 Table. Administrative codes**

| **Comorbidity** | **International Classification of Diseases, 9.0 Clinical Modification codes** |
| --- | --- |
| Cardiac arrest | 427.5 |
| Cardiogenic shock | 785.51 |
| Acute kidney injury | 584, 584.5, 584.6, 584.7, 584.8, 584.9 |
| Respiratory failure | 518.81, 518.82, 518.85, 786.09, 799.1, 96.7, 96.70, 96.71, 96.72 |
| Hepatic failure | 570.0, 572.2, 573.3, 573.4 |
| Hematologic failure | 286.6-286.9, 287.4, 287.5 |
| Neurological failure | 293, 293.0, 293.1, 293.8, 293.81-293.84, 293.89, 293.9, 348.1, 348.3, 348.30, 348.81, 348.39, 780.01, 780.09, 89.14 |
| Invasive hemodynamic assessment | 37.21, 37.23, 204 |
| Coronary angiography | 37.22, 37.23, 88.53-88.56 |
| Percutaneous coronary intervention | 00.66, 36.01, 36.02, 36.05, 36.06, 36.07, 88.57 |
| Intra-aortic balloon pump | 37.61 |
| Percutaneous mechanical circulatory support | 37.68 |
| Extra-corporeal membrane oxygenation | 39.65 |
| Invasive mechanical ventilation | 96.7, 96.70, 96.71, 96.72 |
