# Supplementary material for: Influence of primary payer status on the management and outcomes of ST-segment elevation myocardial infarction in the United States
Source: PLoS One. 2020 Dec 18;15(12):e0243810. doi: 10.1371/journal.pone.0243810 (PMC7748387; doi:10.1371/journal.pone.0243810)
Supplement: S2 Table — (DOCX) [file pone.0243810.s002.docx]

**S2 Table. Predictors of coronary angiography use in STEMI**

| **STEMI admissions** | | **Odds ratio** | **95% confidence interval** | | ***P*** |
| --- | --- | --- | --- | --- | --- |
|  |  |  | **Lower Limit** | **Upper Limit** |  |
| **Insurance type** | **Medicare** | Reference category | | | |
|  | **Medicaid** | 0.93 | 0.91 | 0.94 | <0.001 |
|  | **Private** | 1.25 | 1.24 | 1.26 | <0.001 |
|  | **Uninsured** | 1.09 | 1.07 | 1.10 | <0.001 |
|  | **Others** | 1.14 | 1.12 | 1.16 | <0.001 |
| **Age groups (years)** | **18-49** | Reference category | | | |
|  | **50-59** | 0.92 | 0.91 | 0.93 | <0.001 |
|  | **60-69** | 0.87 | 0.86 | 0.88 | <0.001 |
|  | **70-79** | 0.79 | 0.78 | 0.80 | <0.001 |
|  | **≥80** | 0.32 | 0.32 | 0.32 | <0.001 |
| **Female sex** | | 0.82 | 0.82 | 0.83 | <0.001 |
| **Race** | **White** | Reference category | | | |
|  | **Black** | 0.61 | 0.60 | 0.61 | <0.001 |
|  | **Others** | 0.87 | 0.87 | 0.88 | <0.001 |
| **Charlson comorbidity index** | **0-3** | Reference category | | | |
|  | **4-6** | 0.69 | 0.69 | 0.70 | <0.001 |
|  | **≥7** | 0.42 | 0.41 | 0.42 | <0.001 |
| **Quartile of median household**  **income for zip code** | **0-25^th^** | Reference category | | | |
|  | **26^th^-50^th^** | 0.95 | 0.94 | 0.96 | <0.001 |
|  | **51^st^-75^th^** | 0.92 | 0.91 | 0.93 | <0.001 |
|  | **75^th^-100^th^** | 0.90 | 0.89 | 0.91 | <0.001 |
| **Weekend admission** | | 0.96 | 0.96 | 0.97 | <0.001 |
| **Hospital teaching**  **status and location** | **Rural** | Reference category | | | |
|  | **Urban Non-Teaching** | 4.39 | 4.36 | 4.43 | <0.001 |
|  | **Urban Teaching** | 8.79 | 8.71 | 8.86 | <0.001 |
| **Hospital bed-size** | **Small** | Reference category | | | |
|  | **Medium** | 1.90 | 1.89 | 1.92 | <0.001 |
|  | **Large** | 4.12 | 4.08 | 4.15 | <0.001 |
| **Hospital region** | **Northeast** | Reference category | | | |
|  | **Midwest** | 1.97 | 1.96 | 1.99 | <0.001 |
|  | **South** | 1.73 | 1.71 | 1.74 | <0.001 |
|  | **West** | 1.32 | 1.31 | 1.33 | <0.001 |
| **Tertiles of admissions year** | **2000-2005** | Reference category | | | |
|  | **2006-2011** | 2.15 | 2.14 | 2.16 | <0.001 |
|  | **2012-2017** | 3.93 | 3.90 | 3.96 | <0.001 |
| **STEMI location** | **Anterior** | 3.15 | 3.10 | 3.20 | <0.001 |
|  | **Inferior** | 3.52 | 3.46 | 3.58 | <0.001 |
|  | **Other** | 1.07 | 1.05 | 1.09 | <0.001 |
| **Cardiogenic shock** | | 1.13 | 1.12 | 1.14 | <0.001 |
| **Cardiac arrest** | | 0.68 | 0.67 | 0.68 | <0.001 |
| **Do-not-resuscitate status** | | 0.31 | 0.30 | 0.31 | <0.001 |
| **Palliative care referral** | | 0.30 | 0.30 | 0.31 | <0.001 |
