# Supplementary material for: Influence of primary payer status on the management and outcomes of ST-segment elevation myocardial infarction in the United States
Source: PLoS One. 2020 Dec 18;15(12):e0243810. doi: 10.1371/journal.pone.0243810 (PMC7748387; doi:10.1371/journal.pone.0243810)
Supplement: S3 Table — (DOCX) [file pone.0243810.s003.docx]

**S3 Table. Predictors of in-hospital mortality in STEMI**

| **STEMI admissions** | | | **Odds ratio** | **95% confidence interval** | | ***P*** |
| --- | --- | --- | --- | --- | --- | --- |
|  |  |  |  | **Lower Limit** | **Upper Limit** |  |
| **Insurance type** | | **Medicare** | Reference category | | | |
|  |  | **Medicaid** | 0.96 | 0.94 | 0.99 | 0.002 |
|  |  | **Private** | 0.73 | 0.72 | 0.75 | <0.001 |
|  |  | **Uninsured** | 1.14 | 1.11 | 1.16 | <0.001 |
|  |  | **Others** | 0.91 | 0.88 | 0.94 | <0.001 |
| **Age groups (years)** | | **18-49** | Reference category | | | |
|  |  | **50-59** | 1.39 | 1.36 | 1.43 | <0.001 |
|  |  | **60-69** | 1.90 | 1.86 | 1.95 | <0.001 |
|  |  | **70-79** | 2.86 | 2.79 | 2.94 | <0.001 |
|  |  | **≥80** | 5.05 | 4.92 | 5.19 | <0.001 |
| **Female sex** | | | 1.19 | 1.18 | 1.20 | <0.001 |
| **Race** | | **White** | Reference category | | | |
|  |  | **Black** | 0.97 | 0.95 | 0.99 | 0.002 |
|  |  | **Others** | 1.05 | 1.04 | 1.06 | <0.001 |
| **Charlson comorbidity index** | | **0-3** | Reference category | | | |
|  |  | **4-6** | 1.38 | 1.36 | 1.40 | <0.001 |
|  |  | **≥7** | 1.54 | 1.51 | 1.57 | <0.001 |
| **Quartile of median household**  **income for zip code** | | **0-25^th^** | Reference category | | | |
|  |  | **26^th^-50^th^** | 0.95 | 0.94 | 0.97 | <0.001 |
|  |  | **51^st^-75^th^** | 0.92 | 0.91 | 0.93 | <0.001 |
|  |  | **75^th^-100^th^** | 0.89 | 0.88 | 0.90 | <0.001 |
| **Weekend admission** | | | 1.04 | 1.03 | 1.05 | <0.001 |
| **Hospital teaching**  **status and location** | | **Rural** | Reference category | | | |
|  |  | **Urban Non-Teaching** | 1.12 | 1.11 | 1.14 | <0.001 |
|  |  | **Urban Teaching** | 1.24 | 1.22 | 1.25 | <0.001 |
| **Hospital bed-size** | | **Small** | Reference category | | | |
|  |  | **Medium** | 1.05 | 1.03 | 1.06 | <0.001 |
|  |  | **Large** | 1.13 | 1.11 | 1.14 | <0.001 |
| **Hospital region** | | **Northeast** | Reference category | | | |
|  |  | **Midwest** | 1.05 | 1.03 | 1.06 | <0.001 |
|  |  | **South** | 1.13 | 1.12 | 1.14 | <0.001 |
|  |  | **West** | 0.90 | 0.89 | 0.91 | <0.001 |
| **Tertiles of admissions year** | | **2000-2005** | Reference category | | | |
|  |  | **2006-2011** | 0.74 | 0.74 | 0.75 | <0.001 |
|  |  | **2012-2017** | 0.48 | 0.47 | 0.49 | <0.001 |
| **STEMI location** | | **Anterior** | 1.11 | 1.08 | 1.13 | <0.001 |
|  |  | **Inferior** | 1.00 | 0.98 | 1.02 | 0.99 |
|  |  | **Other** | 1.49 | 1.46 | 1.53 | <0.001 |
| **Cardiogenic shock** | | | 3.98 | 3.93 | 4.03 | <0.001 |
| **Cardiac arrest** | | | 8.60 | 8.51 | 8.70 | <0.001 |
| **Respiratory failure** | | | 1.63 | 1.61 | 1.65 | <0.001 |
| **Acute kidney injury** | | | 2.23 | 2.21 | 2.26 | <0.001 |
| **Systolic heart failure** | | | 0.48 | 0.47 | 0.49 | <0.001 |
| **Prior coronary artery bypass grafting** | | | 0.92 | 0.90 | 0.93 | <0.001 |
| **Coronary angiography** | | | 0.41 | 0.40 | 0.41 | <0.001 |
| **Percutaneous coronary intervention** | | | 0.44 | 0.44 | 0.45 | <0.001 |
| **Coronary artery bypass grafting** | | | 0.43 | 0.43 | 0.44 | <0.001 |
| **Pulmonary artery catheterization** | | | 1.19 | 1.16 | 1.23 | <0.001 |
| **Mechanical circulatory support** | | | 2.05 | 2.02 | 2.08 | <0.001 |
| **Invasive mechanical ventilation** | | | 2.94 | 2.90 | 2.97 | <0.001 |
| **Hemodialysis** | | | 1.32 | 1.27 | 1.37 | <0.001 |
| **In-hospital complications** | **Vascular** | | 0.99 | 0.95 | 1.03 | 0.62 |
|  | **Hemorrhage** | | 0.74 | 0.72 | 0.77 | <0.001 |
|  | **Blood transfusion** | | 0.74 | 0.73 | 0.75 | <0.001 |
|  | **Ischemic stroke** | | 1.41 | 1.38 | 1.45 | <0.001 |
|  | **Intracranial hemorrhage** | | 5.49 | 5.24 | 5.75 | <0.001 |
| **Do-not-resuscitate status** | | | 3.26 | 3.18 | 3.33 | <0.001 |
| **Palliative care referral** | | | 6.57 | 6.41 | 6.73 | <0.001 |
